# First record of mitochondrial genome of *Teredorus nigropennis* (Orthoptera: Tetrigidae) and phylogenetic analysis

**DOI:** 10.1080/23802359.2020.1730259

**Published:** 2020-02-27

**Authors:** Xiao-Dong Li, Yu-Qi Wang, Wei-An Deng, Wan-Tao Rong, Ran Li

**Affiliations:** a School of Chemistry and Bioengineering, Hechi University, Yizhou, PR China;; b The Key Laboratory of Jiangsu Biodiversity and Biotechnology, College of Life Sciences, Nanjing Normal University, Nanjing, PR China

**Keywords:** Orthoptera, Tetrigidae, *Teredorus nigropennis*, mitogenome, phylogenetic analysis

## Abstract

The nearly complete mitochondrial genome (mitogenome) of *Teredorus nigropennis* was determined and analyzed. This mitogenome was 14,652 bp in size and encoded 13 protein-coding genes, two ribosomal RNA genes, 22 transfer RNA genes. The most common start codon is ATN, the most common termination codon is TAA and two genes have incomplete termination codon T (TA). The overall nucleotide composition was 45.2% of A, 10.2% of G, 28.6% of T, and 16.1% of C. The data will increase the basic information of Tetrigidae phylogenetic research and can help to better understand the phylogenetic status of *T*. *nigropennis* in Tetrigiodea.

*Teredorus nigropennis*, belongs to the genus *Teredorus*, within the subfamily Tetriginae of the family Tetrigidae of the order Orthoptera. To date, the genus includes 29 known species worldwide, distributed mainly in South America, China, India and Nepal. (Deng et al. 2014). *T. nigropennis* was described by Deng et al. (2013) from Lin’an, Zhejiang, China. None mitochondrial genome was reported about the genus. In the present study, we sequenced the mitochondrial genome of *T*. *nigropennis* (GenBank accession No. MN938922), which will help to better understand the phylogenetic status of this species in Tetrigidae.

The samples of *T*. *nigropennis* were collected in Wuyishan in Fujian Province, China. The collected specimens were stored in 95% ethanol at temperature −20 °C and deposited in the Museum of Insects of Hechi University (the voucher No. O201). Whole genomic DNA was extracted from legs of adult specimen using a Wizard® Genomic DNA Purification Kit (Promega, Madison, USA) according to the manufacturer’s instructions. The genomic DNA was sequenced using the Illumina Novaseq platform (Personalbio, Shanghai, China). The mitogenome of *Tetrix japonica* (GenBank accession No. JQ340002) was employed as the reference sequence (Xiao et al. 2012). Mitochondrial genome was assembled by Geneious 9.0.4 (https://www.geneious.com), and annotated using MITOS Web Server (Bernt et al. 2013).

We obtained partial mitogenome of *T. nigropennis* with 14,652 bp long. The region that we failed to sequence was between *rrnS* and *trnI*, and generally contained a putative AT-rich region. This mitogenome encoded 13 protein-coding genes (PCGs), 22 tRNAs, 2 ribosomal RNA unit genes (*rrnL* and *rrnS*). The overall nucleotide composition was 45.2% of A, 10.2% of G, 28.6% of T, and 16.1% of C. Ten PCGs started with typical ATN codon (one with ATC, one with ATT, two with ATA, six with ATG), whereas the *ND1*, *ND2*, and *ND6* gene appeared to start with TTA, TTG and GTA, respectively. Eleven PCGs end with complete stop codons (two with TAG, nine with TAA), and the other two genes (*COIII* and *ND5*) end with T (TA) as the incomplete stop codons, which were presumably completed as TAA by post transcriptional polyadenylation (Anderson et al.^1981^). The 22 tRNA genes range in size from 61 to 69 bp. The *rrnS* (744 bp) and *rrnL* (1356 bp), were located between the *trnL1* and AT-rigion, and separated by the *trnV* gene.

To validate the phylogenetic position of *T. nigropennis*, the Bayesian Inference (BI) and Maximum Likelihood (ML) tree was constructed on CIPRES Portal using 13 PCGs from mitogenomes of seven species and one outgroup, respectively. We used the best-fit partitioning scheme and partition-specific models recommended by PartitionFinder (Lanfear et al. 2012). Two phylogenetic analyses using different methods yielded the same topology, and nodal supporting values were always higher for BI tree than for ML tree (Figure 1). As shown in Figure 1, T*. nigropennis* was positioned in the family of Tetriginae, and five species within Tetriginae were grouped in a clade, which suggested Tetriginae was monophyly.

**Figure 1.**
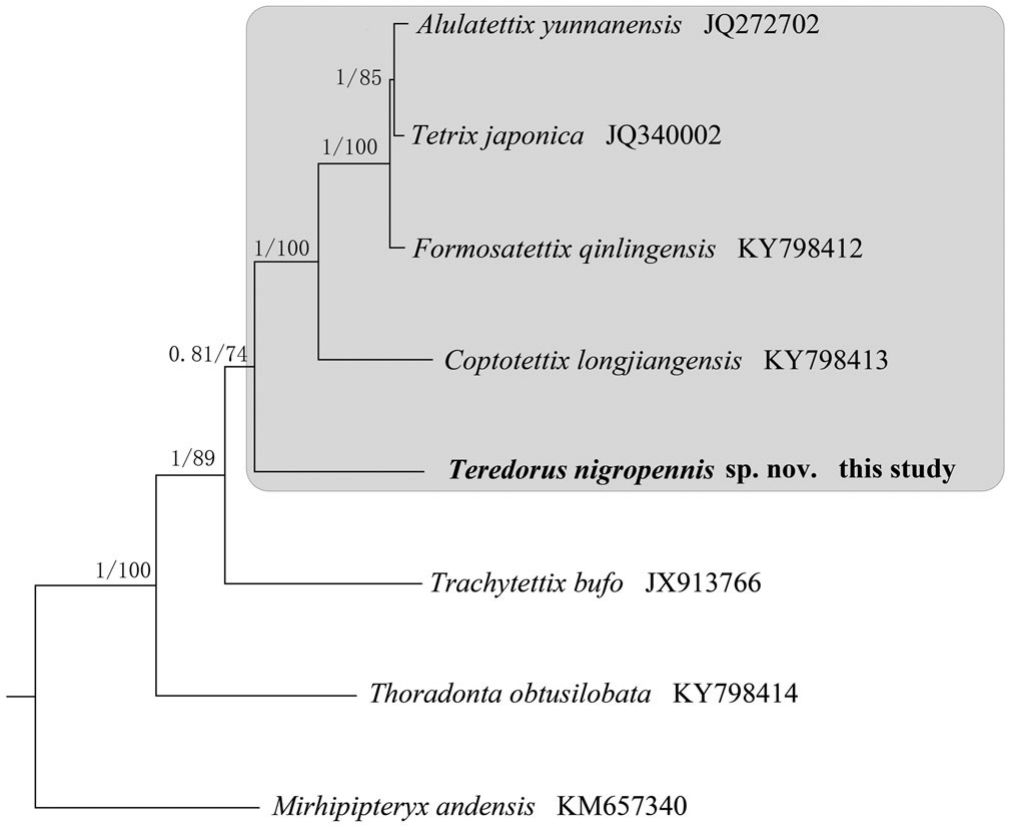
Phylogenetic tree obtained from ML and BI analysis based on 13 concatenated mitochondrial PCGs. Numbers on node are posterior probability (PP) and bootstrap value (BV). Species of Tetriginae are highlighted in gray.
